# Quantitative Analysis of Solubility Parameters and Surface Properties of Larch Bark Proanthocyanidins

**DOI:** 10.3390/polym12122800

**Published:** 2020-11-26

**Authors:** Siqi Chen, Jie Song, Liuping Du, Yanli Ma, Shixue Ren, Junxue Ren, Shujun Li

**Affiliations:** 1 Key Laboratory of Bio-Based Material Science and Technology of Ministry of Education and College of Materials Science and Engineering, Northeast Forestry University, Harbin 150040, China; csq18738668529@163.com (S.C.); sj18846799984@163.com (J.S.); dlp3278583713@163.com (L.D.); myl219@126.com (Y.M.); lishujun@nefu.edu.cn (S.L.); 2 School of Astronautics, Beihang University, Beijing 100191, China; rjx_buaa@163.com

**Keywords:** proanthocyanidins, solubility parameter, inverse gas chromatography, molecular dynamics simulation, surface properties

## Abstract

Quantitative characterization of the solubility parameters and surface properties of larch bark proanthocyanidins will lay the foundation for quantitative studies of the interfacial interactions of proanthocyanidin/polymer composites and will improve the compatibility of components, with important practical and scientific significance. Here, the solubility parameters of highly polymerized larch polymeric proanthocyanidins (LPPCs) and less highly polymerized larch oligomeric proanthocyanidins (LOPCs) were determined experimentally by inverse gas chromatography (IGC). These values were then compared with the solubility parameters obtained using molecular dynamics simulations. The experimentally measured solubility parameters of LPPCs and LOPCs (20.5 and 22.09 (J/m^−3^)^0.5^, respectively) were in good agreement with the solubility parameters determined by molecular dynamics simulations (20.57 and 22.35 (J/m^−3^)^0.5^, respectively. IGC was also used to experimentally determine the total surface energy, which includes the dispersive component of surface energy$\;\gamma_{s}^{d}\;$ and the specific component of surface energy $\gamma_{s}^{sp}$, together with the surface acidity and basicity parameters of LPPCs and LOPCs at different temperatures. The surface properties of proanthocyanidins can be quickly and accurately evaluated by IGC, and both LPPCs and LOPCs were shown to be amphoteric materials. This study provides theoretical and technical support for the use of larch bark proanthocyanidins, which are non-toxic, renewable, and have good ultraviolet resistance, in the field of blending composites. The study also provides a reference for other studies on the interfacial interactions of wood fiber polymer composites.

## 1. Introduction

Larch bark, a by-product of the forestry industry, proanthocyanidins of content as high as 10–16% [1,2], Proanthocyanidins, or condensed tannins, are non-toxic, renewable, and biodegradable polyphenolic compounds with good antibacterial and antioxidant properties and strong resistance to UV light [3,4]. Polymer composites with specific properties can be formed from proanthocyanidins and higher molecular weight polymers, such as polyvinyl alcohol (PVA) [5,6], polylactic acid [7,8], and soybean protein [9,10]. For example, Wang and Wang [10] added valonea tannin to soybean protein to prepare a tannin/soybean protein composite film that had improved mechanical, antioxidant and UV absorption properties, compared with soybean protein. However, at higher pH surface acidity and alkalinity affected the interaction of tannin with the composite network, resulting in a decrease in water vapor permeability and oxygen permeability of the film. In another example, Zhai et al. [11]. prepared a composite membrane from larch tannin extract and PVA. When the composite membrane contained only a small amount of tannin, the strength of the membrane was reduced compared with a PVA membrane but, as the amount of tannin was increased, the strength of the membrane increased because the interfacial interactions between tannin and PVA became stronger. These studies show that the surface properties of tannins can have a large effect on the interfacial interactions and properties of composite polymer materials.

There have been few studies on the surface properties of larch bark proanthocyanidins and their compatibility with other polymers. Quantitative characterization of the surface properties of proanthocyanidins would, therefore, lay the foundation for further quantitative studies of the interfacial effects between the components of proanthocyanidin composites and, importantly, could improve the compatibility of the components. Relating quantitative characterization of interfacial properties, IGC technology has been confirmed by many researchers to be a more accurate, comprehensive and rapid method for studying the properties of solid surfaces.

Inverse gas chromatography (IGC) is a powerful and sensitive analytical technique that uses small molecule probes to provide information about the surface properties of solids [12,13]. IGC provides quantitative information about surface energy, surface dispersion energy, and surface acidity and alkalinity, and has been widely used to investigate the surface properties [14] of different solid forms, including powders [15,16,17], fibers [18,19,20], and resins [21,22]. The Hansen solubility parameter [23,24], which provides a basis for understanding interactions between the components of a composite and improving compatibility between the components, can also be obtained quantitatively using IGC. As part of our research into the utilization of proanthocyanidins in composites with other materials, here, we have determined the solubility parameters and surface properties of larch bark proanthocyanidins using IGC [25,26]. Our results provide a quantitative reference for further studies on interfacial effects in proanthocyanidins-based composites [27].

At the same time, we combined computational simulation to verify the accuracy of the IGC experiment. Molecular dynamics (MD) simulation is a powerful, and now widely used, tool for investigating molecular interaction mechanisms within polymers [28,29] and can accurately predict thermodynamic properties, such as solubility parameters, at a given temperature and pressure [30]. As an example, Gupta et al. [31]. used MD simulations to predict the compatibility of indomethacin with carriers (polyoxyethylene, glucose, and sucrose) and to calculate their cohesive energy density and solubility parameters. As another example, Chang et al. [32]. calculated the solubility parameters of five phosphorus flame retardants using MD simulations, and determined the intermolecular interactions between the flame retardants and polyethylene terephthalate. These studies demonstrate the effectiveness of computer simulations in calculating visual information, such as the solubility parameters of polymers.

Based on the above analysis, we have quantitatively determined the solubility parameters and surface properties of larch bark polymeric proanthocyanidins (LPPCs) and depolymerized oligomeric proanthocyanidins (LOPCs) using IGC. We then combined with molecular dynamics simulation, a standard model of proanthocyanidin trimer and heptamer was established, and the solubility parameters of the two were calculated to verify the accuracy of the IGC experimental results. The IGC method was also used to determine the surface acidity and alkalinity of larch bark proanthocyanidins. And the surface energy was calculated and characterized. The purpose of the research is to provide theoretical support for the design and application of proanthocyanidin-based composite materials, and to provide a useful reference for the quantitative characterization of other polymer composite interface properties.

## 2. Materials and Methods

### 2.1. Reagents

Chromatography grade solvents (*n*-hexane, *n*-heptane, *n*-octane, *n*-nonane, *n*-decane, cyclopentane cyclohexane, dichloromethane, trichloromethane, trichloroethylene, benzene, toluene, *p*-xylene, *o*-xylene, ethanol, 1-propanol, acetone, methyl ethyl ketone, tetrahydrofuran, and ethyl acetate) for IGC were purchased from Shanghai Aladdin Industrial Co., Ltd. (Shanghai, China). Ru/C catalyst was purchased from Kaida Chemical Co., Ltd. (Beijing, China).

#### 2.1.1. Isolation of Larch Bark Polymeric Proanthocyanidins

Larch bark (150 g, particle size 0.5–1.0 mm) was refluxed with aqueous ethanol (70% (*v/v*), 1500 mL) for 3 h and the mixture was then filtered. The solid residue was refluxed with aqueous ethanol (70% (*v/v*), 1000 mL) for 2 h and the mixture was filtered. The filtrates were combined, extracted twice with an equal volume of petroleum ether, and then allowed to stand for 1 h to allow impurities, such as gum and resin, to settle. The purified solution was decanted and the ethanol was removed using a rotary evaporator (bath temperature 45 ± 5 °C). The resulting aqueous solution was filtered through filter paper to remove red insoluble material and extracted six times with an equal volume of ethyl acetate. The aqueous layer was evaporated (55 ± 5 °C) to about 50 mL and then vacuum dried at 50 ± 2 °C to provide LPPCs.

#### 2.1.2. Preparation of Larch Bark Oligomeric Proanthocyanidins

LOPCs were prepared as previously described [33]. Briefly, LPPCs (0.1 g) were dissolved in aqueous ethanol (70% (*v/v*), 20 mL) and hydrogenated for 1 h over Ru/C catalyst (0.001 g), at a constant temperature of 150 °C and a constant hydrogen pressure of 3 MPa, in a Dalian GCF Type 1 high pressure reactor (China First Heavy Industries Co., Ltd., Dalian, China), with a mixing speed of 500 rpm. The catalyst was then removed using a 0.45 μm filter membrane and the filtrate was evaporated. The residue was dried under vacuum at 45 ± 5 °C to provide LOPCs.

### 2.2. Determination of Average Degree of Polymerization of Larch Bark Proanthocyanidins

The ^1^H NMR spectrum of proanthocyanidins (30 mg) in DMSO-d6 (0.6 mL) was recorded using a Bruker AVANCE III HD 500 MHz spectrometer (Brock Scientific Instruments, Beijing, China). The experimental conditions were: Probe, 5 mm BBO; frequency, 600.192 MHz; spectral width, 12,335.5 Hz; pulse width, 13 μs; sampling time, 17 s; delay time, 6 s; sampling times, 16. The average degree of polymerization was calculated using Equation (1) [27,34]

Average degree of polymerization:

(1) $$N\;=\;2\times \frac{A_{6,8}}{A_{e}}\;-1$$

where *A_6,8_* is the total area of integration of the proanthocyanidin C_6_ and C_8_ protons (5.8–6.5 ppm) and *Ae* is the peak area of the hydrogen on the C_4_ equatorial bond of the C-ring of the proanthocyanidin terminal catechin unit (2.5–3.0 ppm).

### 2.3. Inverse Gas Chromatography Was Used to Calculate Solubility Parameters

Alkanes (*n*-hexane, *n*-heptane, *n*-octane, *n*-nonane, *n*-decane, cyclopentane and cyclohexane), halohydrocarbons (dichloromethane, trichloromethane, and trichloroethylene), aromatic hydrocarbons (benzene, toluene, *p*-xylene, and *o*-xylene), alcohols (ethanol and 1-propanol), ketones (acetone and methyl ethyl ketone), ethers (tetrahydrofuran), and esters (ethyl acetate), were used as small molecule probes in the IGC experiments to study the interaction between the polymers and probe solvents with different properties. (The above probe solvents are purchased from Shanghai Aladdin Co., Ltd., all of which are chromatographic grade) Physical property parameters of small molecule probe solvents by IGC are shown in Table A1 (Appendix A).

#### 2.3.1. Determination of Retention Time

Protoanthocyanins from larch bark were dissolved in acetone and 6201 pickling red carrier (60–80 mesh, the supplier is from Guangfu Fine Chemical Research Institute(Tianjin, China)) were added to the solution (volume of the discs was 1–1.2 times that of acetone) at a ratio of 1:10 (*w/w*). The mixture was evenly mixed and then heated in an oven at 50 °C for 24 h. The dried mixture was used to pack a 50 cm stainless steel chromatography column with an inner diameter of 1/8 in. The packed column was then put into an Agilent 6890 N gas chromatograph (Agilent Technologies, Beijing, China), equipped with a thermal conductivity detector. The operating temperature was 180 °C, and 99.999% pure N_2_, at a flow rate of 20 mL/min, was used as the shielding gas. To protect the probe solvents, the column was aged with shielding gas at a flow rate of 20 mL/min for 24 h. After aging, the probe solvent (0.2 μL) was injected onto the column using a Hamilton syringe, and the retention time of the probe solvent was measured at different column temperatures. Each sample was injected three times in parallel and the average value was used for the calculations.

#### 2.3.2. Characteristic Retention Volume of Probe Solvent

The retention time of the probe solvent was obtained by IGC, and the characteristic retention volume $V_{g}^{0}$ of the probe solvent was calculated using Equation (2) [35]

(2) $$V_{g}^{0}\;=\;273.15JF\frac{∆t}{mT}\;$$

where *J* is the non-ideal gas compression factor, *F* is the carrier velocity, Δ*t* is the difference between the retention time tr of the other probe solvent and the retention time tm of n-pentane, *m* is the mass of proanthocyanidins in the fixed phase and *T* is the column temperature. *J* was calculated using Equation (3)

(3) $$J=\frac{3}{2}\frac{{\left(P_{i}/P_{o}\right)}^{2}-1}{{\left(P_{i}/P_{o}\right)}^{3}-1}\;$$

where $P_{i}$ and $P_{o}$ are the inlet and outlet pressures, respectively, of the stainless steel column.

#### 2.3.3. Thermodynamic Parameters of Probe Molecules

Using the molar absorption enthalpy ($\Delta H_{l}^{s}$), the molar mixing enthalpy ($\Delta H_{l}^{\infty }$) at infinite dilution and molar evaporation ($\Delta H_{v}$) of the probe solvent can be calculated using Equations (4)–(6) [36,37]:

(4) $$\Delta H_{l}^{s}=-R\frac{\partial \left(lnV_{g}^{0}\right)}{\partial \left(1/T\right)}$$

(5) $$\Delta H_{l}^{s}=-R\frac{\partial \left(ln\Omega_{1}^{\infty }\right)}{\partial \left(1/T\right)}$$

(6) $$\Delta H_{v}=\Delta H_{l}^{\infty }-\Delta H_{l}^{s}\;$$

where $\Omega_{1}^{\infty \;}$ is the infinite dilution activity coefficient, which can be obtained from Equation (7)

(7) $$ln\Omega_{1}^{\infty }=ln\frac{273.15R}{P_{1}^{0}V_{g}^{0}M_{1}}-\frac{P_{1}^{0}}{RT}\left(B_{11}-V_{1}\right)$$

where *R* is the universal gas constant, *M_1_* is the molar mass of the probe solvent, $P_{1}^{0}$ is the saturated vapor pressure of the probe solvent at temperature *T*, *B_11_* is the coefficient in the second dimension and $V_{1}$ is the molar volume.

#### 2.3.4. Flory–Huggins Interaction Parameter $\chi_{12}^{\infty }$

The Flory–Huggins interaction parameter $\chi_{12}^{\infty }$, which reflects the change in interaction energy caused by the polymer mixing with the solvent [36], can be calculated using Equation (8) [38]:

(8) $$\chi_{12}^{\infty }=ln\frac{273.15R}{P_{1}^{0}V_{g}^{0}M_{1}}-\frac{P_{1}^{0}}{RT}\left(B_{11}-V_{1}\right)-1.$$

#### 2.3.5. Solubility parameter $\delta_{2}$

The solubility parameter $\delta_{1}$ of the probe solvent is expressed by Equation (9):

(9) $$\delta_{1}={\left(\frac{\Delta H_{v}-RT}{V_{1}}\right)}^{\frac{1}{2}}.$$

The solubility parameter $\delta_{1}$ of the probe solvent can then be used to obtain the solubility parameter $\delta_{2}$ of the proanthocyanidins at different temperatures using Equation (10)

(10) $$\frac{\delta_{1}^{2}}{RT}-\frac{\chi_{12}^{\infty }}{V_{1}}=\left(\frac{2\delta_{2}}{RT}\right)\delta_{1}-\left(\frac{\delta_{2}^{2}}{RT}+\frac{\chi_{s}^{\infty }}{V_{1}}\right)$$

where $\chi_{S}^{\infty }$ is the entropy term of the Flory–Huggins interaction parameter.

You can calculate $\frac{\delta_{1}^{2}}{RT}-\frac{\chi_{12}^{\infty }}{V_{1}}$ and $\delta_{1}$. The solubility parameters ($\delta_{2}$) at room temperature (298.15K) can then be obtained by extrapolation.

### 2.4. Molecular Dynamics Simulation Was Used to Calculate Solubility Parameters

#### 2.4.1. Model Establishment and Optimization

**Figure 1:**
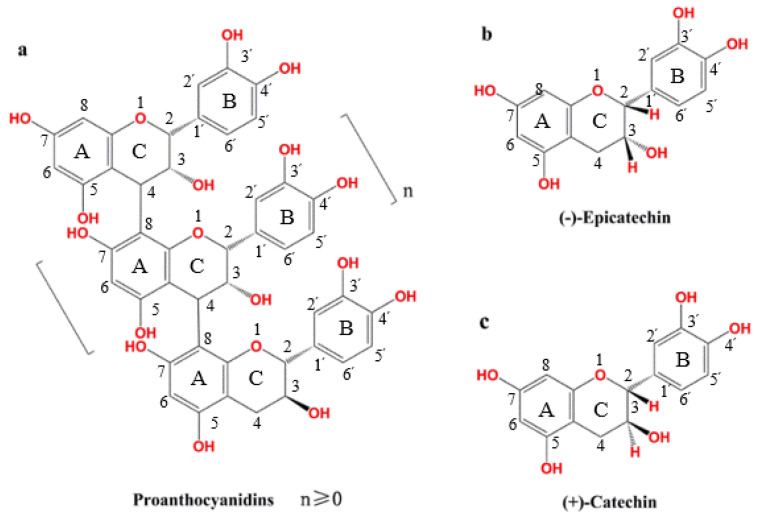
Chemical structure of proanthocyanidins: (**a**) Proanthocyanidins; (**b**) epicatechin; (**c**) catechin.

Proanthocyanidins in larch bark are polymers that have catechin or epicatechin residues as the basic structural units [39,40] (Figure 1). When the degree of polymerization n is ≥5, the polymers are called polymeric proanthocyanidins (PPCs) and, when n is <5, the polymers are called oligomeric proanthocyanidins (OPCs).

The degree of polymerization of larch bark proanthocyanidins was determined using the characteristics of the basic structural units. Two types of ball and stick model, with different degrees of polymerization, were simplified using Chem 3D software. The energy of the models was minimized and geometric optimization was carried out using Material Studio 8.0 (MS 8.0) software.

#### 2.4.2. Calculation of Solubility Parameters

The amorphous cell model, which is a good model for proanthocyanidins, was optimized by setting periodic boundary conditions and optimization of geometry [41]. The Forcite module in Materials Studio 8.0 was used to carry out the MD simulations. The first particle number (N), volume (V), and temperature (T) for the quantitative canonical ensemble (NVT) were calculated. The particle number (N), pressure (P), and temperature (T) for the quantitative constant temperature and constant pressure ensemble (NPT), when the structure system was at equilibrium, were then used to calculate the solubility parameters of proanthocyanidins at 298.15 K. COMPASS II Version 1.2 was chosen as the analogue process force field used by Materials Studio 8.0 software.

### 2.5. Calculation of Surface Properties of Larch Bark Proanthocyanidins

#### 2.5.1. Dispersive Surface Energy

The dispersive surface energy$\;\gamma_{s}^{d}$, which represents the interaction force between the surface of the solid material and the non-polar molecules [42], can be calculated according to the Dorris–Gray method [43], using Equations (11) and (12)

(11) $$\gamma_{s}^{d}=\frac{1}{\gamma_{CH_{2}}}\cdot {\left(\frac{\Delta G_{CH_{2}}}{2Na_{CH_{2}}}\right)}^{2}$$

(12) $$\gamma_{CH_{2}}=36\cdot 8-0.058t$$

where *N* is the Avogadro constant, $∆G_{CH_{2}}\;$ is the increment in adsorption free energy, which is the slope of the linear relationship between $RTlnV_{g}^{0}$, the number of carbon atoms, $\gamma_{CH_{2}}$ is the solid surface energy composed of methylene group and $a_{CH_{2}}$ is the cross-sectional area of the methylene group, which is 0.06 nm^2^ [15].

#### 2.5.2. Specific Surface Energy and Total Surface Energy

The specific component of the surface energy $\gamma_{s}^{sp}$ reflects the surface energy, excluding the dispersive component between the filling proanthocyanidins and the probe [13], but including polarity, acid–base, and hydrogen bond interactions. Theoretically, this can be estimated by the specific adsorption free energy of monopolar probes with different acid–base properties [44]. The values of $\gamma_{l}^{+}$ and $\gamma_{l}^{-}$ for dichloromethane are 124.58 and 0 mJ/m^2^, respectively, and the values of $\gamma_{l}^{+}$ and $\gamma_{l}^{-}$ for toluene are 16.23 and 0 mJ/m^2^ [45], respectively. The specific components of the proanthocyanidins fillers can be calculated using the Good-van Oss method [46], as shown in Equations (13)–(15).

(13) $$\Delta G^{sp}=2Na({\left(\gamma_{l}^{+}\gamma_{s}^{-}\right)}^{1/2}+{\left(\gamma_{l}^{-}\gamma_{s}^{+}\right)}^{1/2})$$

(14) $$\gamma_{s}^{sp}=2{\left(\gamma_{s}^{+}\gamma_{s}^{-}\right)}^{1/2}$$

(15) $$\gamma_{s}=\gamma_{s}^{d}+\gamma_{s}^{sp}$$

where $\gamma_{s}^{+}$ and $\gamma_{s}^{-}$ are, respectively, the contributions to the surface energy of the electron-accepting ability and the electron-donating ability of the surface of the object to be measured, $\gamma_{l}^{+}$ and $\gamma_{l}^{-}$ are, respectively, the contributions to the surface energy of the electron-accepting ability and the electron-donating ability of the probe molecule and $\gamma_{s}$ is the total surface energy.

#### 2.5.3. Surface Acidity and Alkalinity

Lewis acid–base theory was used by Fowkes [47] to analyze the interactions between solids and liquids. The main interactions between solids and liquids are acid–base interactions, which can qualitatively describe the surface acid–base properties of materials and dispersion. The only interaction between the n-alkane probe molecules and the stationary phase is dispersion, and the free energy of adsorption is linear. For some probe molecules there are also acid–base interactions between the probe molecule and the stationary phase, in addition to dispersion. In this case, the free energy of adsorption $\Delta G^{sp}$ deviates from a straight line and the degree of deviation reflects the magnitude of the acid–base interaction between the probe molecule and the surface of the stationary phase [21]. $\Delta G^{sp}$ can be calculated using Equation (16)

(16) $$\Delta G^{sp}=RTlnV_{g}^{0}-RTlnV_{g}^{ref}$$

where $V_{g}^{ref\;}$ is the specific retained volume of a hypothetical n-alkane and a polar probe at the same boiling point. In this study, based on repeated tests, we found that the monopolar probes trichloromethane (acidic probe) and tetrahydrofuran (alkaline probe) and the amphoteric probe ethyl acetate were more suitable for studying the acidity and alkalinity of the proanthocyanidin surface. These three probes, together with n-alkanes, were thus used to calculate $\Delta G^{sp}$.

## 3. Results and Discussion

### 3.1. Average Degree of Polymerization of Proanthocyanidins

**Figure 2:**
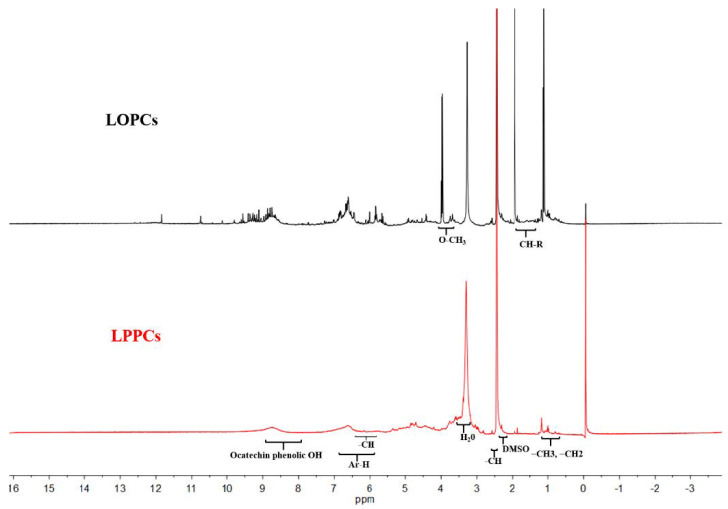
^1^H NMR spectra of larch polymeric proanthocyanidins (LPPCs) and larch oligomeric proanthocyanidins (LOPCs).

The presence of peaks at 0.8–1.2 ppm (methyl), 6.0–7.0 ppm (Ar-H) and 8.0–9.0 ppm (catechol) in the ^1^H NMR spectra of LPPCs and LOPCs (Figure 2) indicate that both are polyphenols.

In solutions with the same concentration, the intensities of the peaks at 1.0 ppm (-CH_2_, -CH_3_ groups) and 8.0–9.0 ppm (catechin hydroxyl group) in the spectrum of LOPCs are higher than those in the spectrum of LPPCs, indicating that LOPCs contains a higher number of methyl, methylene, and catechin hydroxyl groups and thus has a lower degree of polymerization.

The peaks for hydrogens at the C_6_ and C_8_ positions (are shown in Figure 1) of the proanthocyanidins are located at 5.8–6.5 ppm and the integrated peak area was recorded as A_6,8_. The peak for hydrogens at the C_4_ position (is shown in Figure 1) of the C-ring of the terminal catechin subunit is located at 2.5–3.0 ppm and the integrated peak area was recorded as Ae. The average degree of polymerization of LOPCs was 3.26 and that of LPPCs was 7.28. These average degrees of polymerization were used in the MD simulations.

### 3.2. Solubility Parameters of Larch Bark Proanthocyanidins

#### 3.2.1. Retention Volumes of Probe Solvents (by IGC)

Using IGC, the retention volumes of the probe solvents $V_{g}^{0}$ can be determined from Equation (2). Values of $V_{g}^{0}$ are shown in Table A2.

The specific retention volumes of both LPPCs and LOPCs decreased with increasing temperature in the same probe solvent, indicating that increased temperature weakens the interaction between the probe solvent and the proanthocyanidins and reduces the interaction time.

For congeners of alkanes, halogenated hydrocarbons, aromatic hydrocarbons and ketones, as the molecular weight of the probe solvent increases, the retention time between the probe solvent and the proanthocyanidins increases, the value of proanthocyanidins $V_{g}^{0}$ increases, and molecular entanglement between the probe solvent and the proanthocyanidins occurs more easily, increasing the strength of the interaction between the two. However, for alcohols (ethanol and 1-propanol) $V_{g}^{0}$ decreases with increasing molecular weight, indicating that the polar proanthocyanidins interact more strongly with ethanol, the more polar probe solvent. Intermolecular hydrogen bonds increase the strength of the interaction between the two, leading to increased retention time and larger retained volume. Tetrahydrofuran and ethyl acetate, which are a representative ether and ester, are also polar probes. They are, however, less polar than ethanol and have shorter interaction times with the proanthocyanidins, leading to smaller values of $V_{g}^{0}$.

The value of $V_{g}^{0}$ for LOPCs is larger than that for LPPCs at the same temperature with the same probe solvent. This is because LOPCs have a longer interaction time and larger retention volume with the probe solvent and indicates that LOPCs are more compatible with these probe solvents.

#### 3.2.2. Thermodynamic Parameters of Probe Solvents (by IGC)

The thermodynamic parameters of the probe molecules, such as molar enthalpy of absorption $∆H_{l}^{s}$, enthalpy of mixing at infinite molar dilution $∆H_{l}^{\infty }$ and molar enthalpy of evaporation $∆H_{v}$, can be obtained using Equations (4)–(6). The thermodynamic parameters of the probe molecules are shown in Table A3.

Proanthocyanidins are solids and cannot be vaporized, which means they have no enthalpy of evaporation. However, the change in thermodynamic parameters during the adsorption of the probe solvent onto the proanthocyanidins can be indirectly determined by the vaporizable probe solvent and used to infer the interaction between the proanthocyanidins and the probe solvent.

The molar absorption enthalpy $∆H_{l}^{s}$ and molar mixing enthalpy $∆H_{l}^{\infty }$ of the probe solvents are both negative (Table A3), indicating that the adsorption of the probe solvent onto the proanthocyanidins is exothermic. The molar enthalpy of evaporation $∆H_{v}$ of the probe solvents is positive, indicating that evaporation of the probe solvent from the proanthocyanidins is endothermic. For homologous probe solvents, the absolute value of molar absorption enthalpy $∆H_{l}^{s}$ increases with increasing molecular weight, and the adsorption of the probe solvents by the proanthocyanidins is enhanced.

There is no obvious rule to follow when comparing the thermodynamic parameters of the probe solvents for LOPCs and LPPCs. but, on the whole, the molar enthalpy of evaporation $∆H_{v}$ of LOPCs is larger, and the energy required to evaporate the probe solvent from LOPCs is higher, which further indicates that the solubility of the LOPCs in the probe solvent is better.

#### 3.2.3. Flory–Huggins Interaction Parameter $\chi_{12}^{\infty }$ and Infinite Dilution Activity Coefficient $\Omega_{1\;}^{\infty }\;$ (by IGC)

The Flory–Huggins interaction parameter $\chi_{12}^{\infty }$ reflects the change in interaction energy when the proanthocyanidins is mixed with the probe solvent and can be calculated using Equation (8). The smaller the value, the greater the solubility of the proanthocyanidins in the probe solvent. Flory–Huggins interaction parameters are shown in Table A4.

The relationship between $\chi_{12}^{\infty }$ and temperature is not linear (Table A4). According to the Flory Huggins theory, when$\;\chi_{12}^{\infty }$ is > 1, the probe solvent and polymer are not compatible; when $\chi_{12}^{\infty }$ is < 0.5, the probe solvent is a good solvent for the polymer. Only tetrahydrofuran has $\chi_{12}^{\infty }$ < 0.5 at low temperatures, indicating that tetrahydrofuran is a good solvent for proanthocyanidins; the remaining probe solvents have $\chi_{12}^{\infty }$ > 1, indicating that the interactions between the proanthocyanidins and probe solvents are weak.

The values of $\chi_{12}^{\infty }$ for both LPPCs and LOPCs were smaller for nonpolar probes (e.g., alkanes) compared with the values of $\chi_{12}^{\infty }$ for polar probes (e.g., halogenated hydrocarbons, aromatics, alcohols, ketones, esters, and ethers), indicating that LPPCs and LOPCs have better interactions with polar probes and that polar probes are more likely to solubilize proanthocyanidins. The smaller values of $\chi_{12}^{\infty }$ for LOPCs compared with LPPCs also indicate that the depolymerized proanthocyanidins are better solubilized in the probe solvents.

The infinite dilution activity coefficient $\Omega_{1}^{\infty }$, which can be calculated using Equation (7), also reflects the difference in degree of interaction between the proanthocyanidins and probe solvents. The smaller the value of $\Omega_{1}^{\infty }$*,* the greater the solubility of the proanthocyanidin in the probe solvent. Values of $\Omega_{1}^{\infty }$ are shown in Table A5. When $\Omega_{1}^{\infty }$ > 10, the probe is a poor solvent and when $\Omega_{1}^{\infty }$ < 5, the probe is a good solvent [13]. The information reflected by a value in Table A5 is consistent with $\chi_{12}^{\infty }$.

#### 3.2.4. Solubility Parameter $\delta_{2}$ (by IGC)

**Figure 3:**
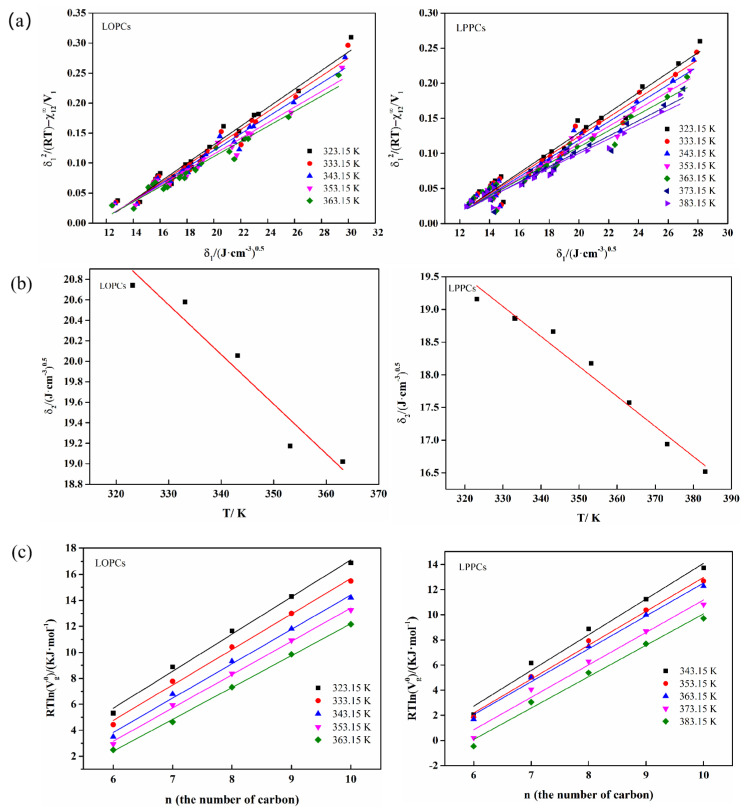
(**a**) Curves of $\frac{\delta_{1}^{2}}{RT}-\frac{\chi_{12}^{\infty }}{V_{1}}$ vs. $\delta_{1}$ at different temperatures for polymeric proanthocyanidins and oligomeric proanthocyanidins; (**b**) Extrapolation to calculate solubility parameter $\delta_{2}$ of proanthocyanidins at 298.15 K; (**c**) Variation of $RTlnV_{N}$ with number of carbon atoms in n-alkanes at different temperatures.

The solubility parameters of the proanthocyanidins at various temperatures can be obtained from the slope of $\frac{\delta_{1}^{2}}{RT}-\frac{\chi_{12}^{\infty }}{V_{1}}$ and $\delta_{1}$, as shown in Figure 3a. The solubility parameters of LOPCs and LPPCs at 298.15 K were calculated to be 22.09 (J/cm^−3^)^0.5^ and 20.5 (J/cm^−3^)^0.5^, respectively, by extrapolation as shown in Figure 3b (Detailed values are shown in Table A6).

### 3.3. Molecular Dynamics Calculation

The compatibility between the components of a polymer blend system has a huge influence on the performance of the blend. The solubility parameter $\delta_{2}$ can be used to quantitatively describe the compatibility of the components of the blend material and to predict the compatibility of blend components when considering a new blend system. In this section, the solubility parameters of LOPCs and LPPCs were calculated using MD simulations and compared with the experimental measurements obtained by IGC, with the expectation that solubility parameters can be obtained from the calculations to avoid the tedious IGC experiments.

#### 3.3.1. Molecular Modeling and Optimization

**Figure 4:**
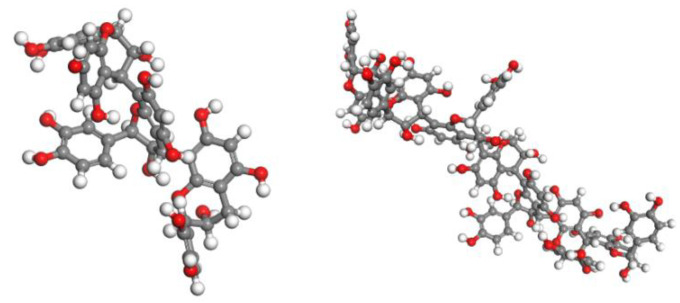
Optimized structures of proanthocyanidins (left, trimer; right, heptamer).

The average degrees of polymerization of LOPCs and LPPCs were experimentally measured as 3.26 and 7.28, respectively. It is thus reasonable to assume that the oligomeric proanthocyanidins and polymeric proanthocyanidins are trimers and heptamers, respectively. We used Chem3D software to establish ball and stick models and carried out energy minimizations. The Smart algorithm of Materials Studio 8.0 software was then used for geometric optimization. The optimized structures, obtained after 10,000 iterations to reach equilibrium, are shown in Figure 4.

#### 3.3.2. Calculation of Solubility Parameters $\delta_{2}$ (by MD)

**Figure 5:**
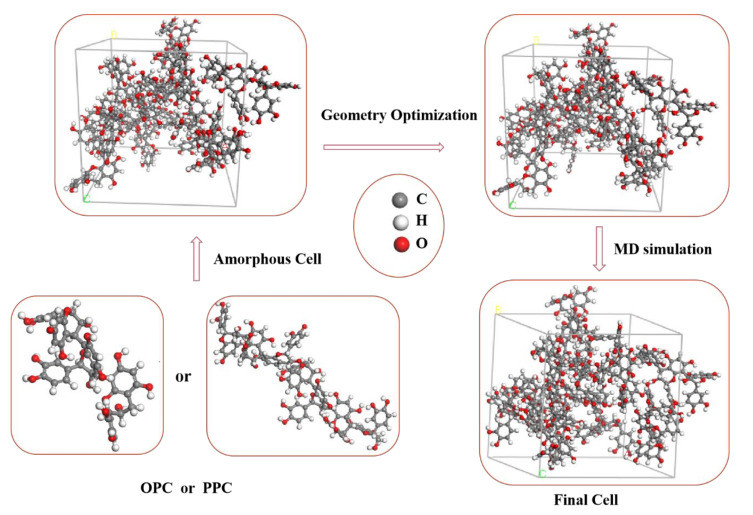
Flow chart showing molecular dynamics simulation (using trimer as an example).

**Figure 6:**
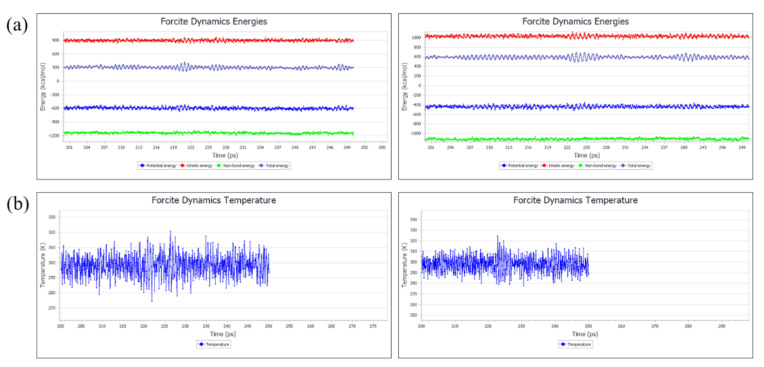
(**a**) Energy fluctuation curves of proanthocyanidins at NVT-MD simulation time (left, trimer; right, heptamer); (**b**) temperature fluctuation curves of proanthocyanidins at NVT-MD simulation time (left, trimer; right, heptamer).

Because of limited computational resources, the optimized structures of the proanthocyanidins were used to create an amorphous model system, consisting of ten trimeric chains and five heptameric chains, using the amorphous cell method. Following optimization of geometry, the optimized amorphous cell system was used in MD simulations to obtain an equilibrium morphology model to calculate solubility parameters (see Figure 5 for flow diagram). Firstly, the NVT ensemble MD simulation was carried out at 298.15 K, using the nose temperature control method. The trimer and heptamer were prerelaxed for 50 ps and 100 ps in steps of 1 fs to release any possible tension in the structures. The NPT simulation was then carried out, and the equilibrium structure of the proanthocyanidins was obtained using the Andersen temperature control method, with a step size of 200 ps. Finally, a 50 ps NVT simulation with 1 fs steps was performed at 298.15 K to obtain the energy fluctuation curve (Figure 6a) and temperature fluctuation curve (Figure 6b). As can be seen in Figure 6, the energy of the proanthocyanidin system, as well as the temperature, fluctuate by less than 10%, indicating that each system has reached an equilibrium state. The Forcite module was then used to analyze the trajectory information in order to perform cohesive energy density calculations. A solubility parameter at 298.15 K was obtained for both the trimer and heptamer (Table A7). The van der Waals force superposition for all NVTs and NPTs was set to atom-based, with a cut-off distance of 15.5 Å, and the electrostatic interaction superposition was set to Ewald, with a precision of 10^−4^ kcal/mol.

### 3.4. Comparison of Solubility Parameters of Larch Bark Polymeric Proanthocyanidins and Oligomeric Proanthocyanidins Obtained by Experimental Inverse Gas Chromatography and Molecular Dynamics Simulations

The solubility parameters of LPPCs and LOPCs, obtained by molecular dynamics simulation, are 20.57 and 22.35 (J·cm^−3^)^0.5^, respectively (Table A7) and the solubility parameters of LPPCs and LOPCs, determined by IGC, are 20.50 and 22.09 (J·cm^−3^)^0.5^, respectively (Table A6). The solubility parameters obtained by the two methods are thus very close to each other, indicating that MD simulations can be used to calculate solubility parameters for proanthocyanidins.

### 3.5. Determination of Surface Properties of Larch Bark Proanthocyanidins by Inverse Gas Chromatography

IGC can be used for the rapid and accurate determination of total surface energy (including dispersive ${\;\gamma }_{s}^{d}\;$ and specific $\;\gamma_{s}^{sp}$ components), as well as surface acidity and alkalinity, of solid materials and has been widely used to quantitatively characterize the surface properties of solids.

#### 3.5.1. Dispersive Component $\gamma_{s}^{d}$ and Specific Component $\gamma_{s}^{sp}$

**Figure 7:**
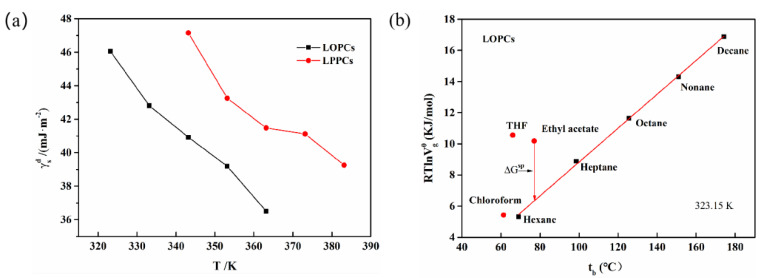
(**a**) Dispersion components of LPPCs and LOPCs at different temperatures; (**b**) Sawyer method: Relationship with probe solvent boiling point t_b_ (at 323.15 K, for LOPCs).

The total surface energy $\gamma_{s}$ of solid materials includes the dispersion component $\gamma_{s}^{d}\;$ and the specific component $\;\gamma_{s}^{sp}$. The dispersion component $\;\gamma_{s}^{d}$ can be calculated using Equations (11) and (12), where the increment in adsorption free energy $∆G_{CH_{2}}$ in the Equation is the slope of the linear relationship between $RTlnV_{g}^{0}$ and the number of carbon atoms, as shown in Figure 3c. The dispersion components of LPPCs and LOPCs vary more regularly with temperature, both decreasing with increasing temperature (Figure 7a). Since the dispersion component indicates the interaction between the surface of solid materials and non-polar molecules, when studying the blending of proanthocyanidins with other polymers, the temperature can be controlled to improve the compatibility of the component surfaces/interfaces and thus improve the performance of the blended polymers.

It can also be seen from Figure 7a that the dispersion component $\gamma_{s}^{d}$ of LOPCs is lower than that of LPPCs at the same temperature. Since the solid surface dispersion component is influenced by non-polar functional groups, the lower $\gamma_{s}^{d}$ of LOPCs may reflect a reduction in non-polar groups in LOPCs after depolymerization of LPPCs.

The specific component $\gamma_{s}^{sp}$, which includes the Lewis acid component $\gamma^{+}$ and the Lewis base component $\gamma^{-}$, can be calculated using Equations (13) and (14). The total surface energy $\gamma_{s}$ can be calculated from $\gamma_{s}^{d}$ and $\;\gamma_{s}^{sp}$ using Equation (15). Total surface energies are shown in Table A8. Both LOPCs and LPPCs contribute less to the total surface energy; the total surface energy of LOPCs is slightly lower than that of LPPCs at the same temperature. This may be because the depolymerization reaction breaks linkages between monomers, leading to an increase in non-polar functional groups on the small molecules, increased interactions and folding between small molecules, which reduces the number of exposed active hydroxyl groups. Since these cover the high-energy positions on the surface of LOPCs, the surface free energy of the LOPCs decreases.

#### 3.5.2. Surface Acidity and Alkalinity

Sawyer’s method [48,49] suggests that the only force between an n-alkane probe molecule and the stationary phase is a dispersive force, in which case the plot of $RTlnV_{g}^{0}$ against the boiling point of the probe solvent $t_{b}$ is a straight line. When there is also an acid–base force between a probe molecule and the stationary phase, the degree of deviation from a straight line reflects the magnitude of the acid–base force between the probe molecule and the surface of the stationary phase. The distance between the polar probe solvent and the reference line for n-alkanes is the surface characteristic adsorption free energy $∆G^{sp}$ (Figure 7b). Values of $∆G^{sp}$ for trichloromethane (acidic probe), tetrahydrofuran (basic probe) and ethyl acetate (amphoteric probe) were calculated using Equation (16) and are shown in Table A9.

The $∆G^{sp}$ value can be calculated using Figure 7b, which shows the relationship between $RTlnV_{g}^{0}$ for LOPCs and the probe solvent boiling point tb at 323.15 K. The monopolar probes represented by trichloromethane (acidic probe) and tetrahydrofuran (basic probe) are both located above the line, with positive $∆G^{sp}$ values, indicating that, at 323.15 K, the solid surfaces of the LOPCs and LPPCs have amphoteric characteristics, with both electron pair-accepting Lewis acid sites and an electron pair-donating Lewis base sites. Ethyl acetate (amphoteric probe) is also located above the straight line, with a certain degree of deviation, further confirming its acid–base amphoteric characteristics. $∆G^{sp}$ values for LPPCs and LOPCs at different temperatures are shown in Table A9. Although, under the same conditions, both LPPCs and LOPCs deviate from the straight line to a certain extent for acidic, basic and amphoteric probes, the $∆G^{sp}$ of tetrahydrofuran (basic probe) is larger than that of trichloromethane (acidic probe). The stronger interaction with tetrahydrofuran (basic probe) and weaker interaction with trichloromethane (acidic probe) indicate that both LOPCs and LPPCs are amphiphilic materials. The $∆G^{sp}$ value of LOPCs was smaller than that of LPPCs at the same temperature, indicating that the activity of the LOPCs acid–base sites was weaker than those of LPPCs.

## 4. Conclusions

In this paper, the solubility parameters of LOPC and LPPC at room temperature are obtained by IGC experimental method and molecular dynamics simulation method. The two data results are consistent, and the solubility parameters of LOPC are larger; At the same time, the surface properties of proanthocyanidins before and after polymerization were characterized by IGC technique. The specific component of surface energy (determined by acid probe “trichloromethane” and basic probe “tetrahydrofuran”) and the total surface energy of proanthocyanidins were calculated; By studying the degree of deviation of the ${“t}_{b}{-RTlnV}_{g}^{0}”$ straight line between the unipolar probe solvent and the n-alkane, $∆G^{sp}$, the changes in the surface acid–base properties of proanthocyanidins before and after degradation were characterized. The above research can provide reference for the selection of solvent for proanthocyanidins from larch bark, and provide more accurate information for the study of interface interaction of proanthocyanidins based composites. However, the quantitative study of proanthocyanidins based composites is only a starting point, and the interface interaction model between proanthocyanidins and polymer components should be further developed to fully clarify the hypothesis of “component surface property–interfacial interaction–performance“. This will provide a theoretical basis for the customized design, efficient preparation and targeting properties of the composites blended with proanthocyanidins and other polymers.

## Appendix A

**Table A1:** Physical property parameters of small molecule probe solvents by inverse gas chromatography (IGC).

Probe Solvent	Number of Carbon	Molecular Mass (g/mol)	Boiling Point (℃)
*n*-hexane	6	86.18	69
*n*-heptane	7	100.2	98.5
*n*-octane	8	114.23	125.6
*n*-nonane	9	128.26	151
*n*-decane	10	142.29	174.2
cyclopentane	5	70.14	49.3
cyclohexane	6	84.16	80.7
dichloromethane	1	84.93	39.8
trichloromethane	1	119.38	61.3
trichloroethylene	2	131.39	87.1
benzene	6	78.11	80
toluene	7	92.14	110.6
*p*-xylene	8	106.167	138.37
*o*-xylene	8	106.16	144.4
ethanol	2	46.07	78
1-propanol	3	60.1	97.1
acetone	2	58.08	56.53
methyl ethyl ketone	3	72.11	79.6
ethyl acetate	4	88.11	77
tetrahydrofuran	4	72.11	66

**Table A2:** Specific retention volumes $V_{g}^{0}$ at different temperatures (mL/g).

Probe Solvent	LPPCs							LOPCs				
	323.15 K	333.15 K	343.15 K	353.15 K	363.15 K	373.15 K	383.15 K	323.15 K	333.15 K	343.15 K	353.15 K	363.15 K
*n*-hexane	5.78	3.78	2.06	1.88	1.75	1.07	0.86	7.23	4.96	3.40	2.71	2.29
*n*-heptane	19.56	12.69	8.61	5.63	5.24	3.71	2.66	27.26	16.58	10.81	7.55	4.66
*n*-octane	54.12	35.25	22.47	14.84	11.94	7.58	5.64	76.34	42.98	26.13	17.33	11.34
*n*-nonane	141.08	87.16	51.4	34.14	27.19	16.44	11.74	205.31	109.09	62.88	41.43	26.16
*n*-decane	367.61	215.97	122.19	75.08	58.37	32.55	22.47	536.47	266.87	145.58	91.39	56.16
cyclopentane	0.71	0.48	0.37	0.27	0.19	0.16	0.31	1.77	1.47	1.20	0.97	0.55
cyclohexane	6.99	4.55	3.18	2.06	1.94	1.56	1.72	9.43	6.63	4.81	3.09	2.01
dichloromethane	2.64	2.13	1.21	0.89	0.49	0.33	0.54	3.66	2.52	1.70	1.06	0.82
trichloromethane	6.99	5.42	3.46	2.5	1.94	1.15	0.94	7.54	5.47	4.00	2.41	2.20
trichloroethylene	13.89	9.78	6.35	4.29	4.08	2.63	2.35	19.3	11.68	7.61	5.02	4.30
benzene	24.12	14.04	8.37	6.14	4.65	2.88	2.43	13.74	11.74	7.49	5.68	4.12
toluene	45.41	24.79	16.19	11.22	9.01	5.67	4.38	41.99	27.88	18.37	12.04	8.60
*p*-xylene	121.83	75.83	42.79	28.85	21.3	12.33	10.02	121.99	75.66	47.71	30.14	20.21
*o*-xylene	184.66	87.45	49.86	35.26	25.47	14.71	13.31	153.57	90.23	56.3	36.69	24.24
ethanol	78.45	42.27	27.84	14.84	11.14	5.18	3.91	103.10	71.89	31.06	16.08	11.07
1-propanol	69.12	28.41	19.31	10.36	5.91	3.21	2.04	66.08	42.39	27.78	9.73	7.41
acetone	27.77	22.95	11.63	8.19	4.74	2.96	1.33	35.36	18.84	14.04	6.93	5.31
methyl ethyl ketone	43.18	33.03	20.37	10.33	7.94	4.69	2.58	67.51	46.89	23.99	13.10	8.05
ethyl acetate	34.87	27.99	26.51	17.99	6.68	3.45	2.51	44.2	28.05	20.51	11.65	7.04
tetrahydrofuran	41.45	28.09	20.65	11.13	6.56	3.86	3.04	51.04	33.71	23.10	11.94	7.77

**Table A3:** Thermodynamic parameters of probe solvents (kJ/mol).

Probe Solvent	LPPCs			LOPCs		
	**$\Delta H_{l}^{s}$**	$\Delta H_{l}^{\infty }$	$\Delta H_{v}$	$\Delta H_{l}^{s}$	$\Delta H_{l}^{\infty }$	$\Delta H_{v}$
*n*-hexane	−31.01	-4.94	26.07	−28.51	−3.23	25.29
*n*-heptane	−32.99	−3.31	29.68	−42.13	−2.12	40.01
*n*-octane	−38.65	−2.21	36.43	−46.15	−1.35	44.8
*n*-nonane	−42.12	−1.47	40.65	−49.75	−0.83	48.92
*n*-decane	−47.54	−0.99	46.56	−54.62	−0.51	54.11
cyclopentane	−29.81	−5.31	24.5	−26.79	−3.53	23.26
cyclohexane	−30.26	−3.62	26.63	−37.49	−2.37	35.11
dichloromethane	−43.24	−4.70	38.53	−37.59	−1.89	35.7
trichloromethane	−35.62	−3.36	32.26	−32.12	−1.43	30.69
trichloroethylene	−31.05	−2.28	28.77	−37.72	−1.08	36.64
benzene	−39.34	−3.34	35.99	−30.53	−2.14	28.4
toluene	−40.95	−2.27	38.68	−39.15	−1.4	37.75
*p*-xylene	−43.61	−1.53	42.07	−44.07	−0.89	43.18
*o*-xylene	−44.9	−1.36	43.54	−44.85	−0.77	44.09
ethanol	−51.89	−2.26	49.64	−58.15	−1.33	56.82
1-propanol	−59.35	−1.81	57.54	−56.94	−1.00	55.93
acetone	−44.57	−3.63	40.94	−46.82	−2.35	44.48
methyl ethyl ketone	−48.73	−2.9	45.84	−53.85	−1.85	52.00
ethyl acetate	−48.95	−3.35	45.59	−44.28	−2.13	42.16
tetrahydrofuran	−47.56	−11.26	36.31	−46.72	−7.68	39.05

**Table A4:** Flory–Huggins interaction parameters $\chi_{12}^{\infty }$ of probe solvents with proanthocyanidins at different temperatures.

Probe Solvent	LPPCs							LOPCs				
	323.1 K	333.15 K	343.15 K	353.15 K	363.15 K	373.15 K	383.15 K	323.15 K	333.15 K	343.15 K	353.15 K	363.15 K
*n*-hexane	3.47	3.56	3.85	3.66	3.46	3.7	3.69	3.24	73.28	3.35	3.29	3.19
*n*-heptane	3.13	3.18	3.2	3.29	3.05	3.11	3.17	2.8	2.91	2.98	3	3.17
*n*-octane	3.01	3.00	3.04	3.07	2.93	3.06	3.05	2.67	2.8	2.89	2.92	2.99
*n*-nonane	2.69	2.94	3.01	2.99	2.82	2.96	2.95	2.58	2.72	2.81	2.8	2.86
*n*-decane	2.92	2.89	2.95	2.96	2.77	2.95	2.94	2.54	2.68	2.78	2.77	2.81
cyclopentane	5.13	5.21	5.18	5.25	5.33	5.27	4.41	4.21	4.09	4.01	3.97	4.29
cyclohexane	3.69	3.77	3.8	3.93	3.71	3.66	3.32	3.39	3.39	3.39	3.53	3.67
dichloromethane	3.29	3.19	3.46	3.5	3.86	4.02	3.3	2.96	3.02	3.12	3.33	3.33
trichloromethane	2.7	2.63	2.77	2.81	2.8	3.07	3.04	2.63	2.62	2.62	2.85	2.67
trichloroethylene	2.78	2.77	2.85	2.93	2.68	2.84	2.69	2.46	2.59	2.67	2.77	2.63
benzene	2.53	2.71	2.89	2.89	2.88	3.09	3.01	3.09	2.89	3	2.97	3
toluene	2.77	2.97	3.02	3.04	2.93	3.09	3.06	2.85	2.86	2.9	2.97	2.98
*p*-xylene	2.71	2.72	2.87	2.87	2.81	3.01	2.9	2.71	2.73	2.76	2.83	2.86
*o*-xylene	2.53	2.81	2.94	2.88	2.83	3.03	2.8	2.72	2.78	2.82	2.84	2.88
ethanol	2.07	2.22	2.21	2.44	2.36	2.78	2.73	1.79	1.69	2.1	2.36	2.36
1-propanol	2.84	3.21	3.12	3.3	3.45	3.68	3.77	2.88	2.81	2.76	3.36	3.22
acetone	1.87	1.72	2.09	2.15	2.42	2.65	3.21	1.63	1.92	1.9	2.32	2.31
methyl ethyl ketone	2.03	1.93	2.06	2.42	2.38	2.63	2.97	1.59	13.58	1.9	2.18	2.37
ethyl acetate	1.98	1.82	1.53	1.6	2.29	2.67	2.73	1.74	1.82	1.79	2.03	2.23
tetrahydrofuran	0.08	0.2	0.26	0.65	0.97	1.31	1.38	−0.13	0.01	0.15	0.58	0.8

**Table A5:** Infinite dilution activity coefficients $\Omega_{1}^{\infty }$ of probe solvents at different temperatures.

Probe Solvent	LPPCs							LOPCs				
	323.15 K	333.15 K	343.15 K	353.15 K	363.15 K	373.15 K	383.15 K	323.15 K	333.15 K	343.15 K	353.15 K	363.15 K
*n*-hexane	87.26	95.32	127.97	105.2	86.29	109.85	108.39	69.7	72.53	77.44	72.84	66.03
*n*-heptane	62.42	65.19	66.93	73.26	57.62	61	64.89	44.78	49.88	53.32	54.63	64.78
*n*-octane	55.19	54.42	56.65	58.65	51.15	57.97	57.35	39.13	44.63	48.72	50.21	53.87
*n*-nonane	52.35	51.5	55.1	54.13	45.72	52.27	51.86	35.97	41.15	45.05	44.61	47.52
*n*-decane	50.19	49.06	51.96	52.63	43.6	51.9	51.33	34.39	39.71	43.62	43.24	45.31
cyclopentane	458.92	495.39	483.87	518.33	560.58	527.76	224.26	183.44	162.88	150.44	144	198.4
cyclohexane	108.99	117.7	121.69	138.74	110.72	105.84	75.09	80.83	80.84	80.4	92.35	106.87
dichloromethane	72.66	65.84	86.61	90	129.18	151.36	73.64	52.3	55.56	61.77	75.77	76.2
trichloromethane	40.53	37.57	43.41	45.09	44.56	58.74	57.07	37.57	37.25	37.46	46.76	39.43
trichloroethylene	44.01	43.19	47.19	50.71	39.56	46.39	40	31.66	36.18	39.38	43.35	37.53
benzene	34.01	40.72	48.88	48.82	48.33	59.65	55	59.71	48.72	54.67	52.79	54.58
toluene	43.55	53.11	55.77	56.65	50.88	59.54	57.9	47.09	47.24	49.16	52.8	53.3
*p*-xylene	40.71	41.45	48.1	48.13	45.15	55.37	49.44	40.66	41.55	43.15	46.07	47.58
*o*-xylene	34.24	45.25	51.37	48.47	46.03	56.02	44.55	41.18	43.86	45.5	46.58	48.36
ethanol	21.47	25.11	24.83	31.27	28.72	43.66	41.78	16.34	14.77	22.26	28.85	28.9
1-propanol	46.36	67.4	61.55	73.67	85.6	107.48	118.39	48.49	45.17	42.79	78.48	68.24
acetone	17.61	15.2	21.95	23.3	30.71	38.29	67.35	13.83	18.52	18.18	27.53	27.47
methyl ethyl ketone	20.77	18.67	21.39	30.56	29.47	37.79	52.84	13.28	13.15	18.16	24.11	29.07
ethyl acetate	19.61	16.8	12.54	13.4	26.75	39.21	41.72	15.47	16.76	16.21	20.68	25.38
tetrahydrofuran	2.93	3.31	3.52	5.21	7.18	10.08	10.76	2.38	2.76	3.15	4.86	6.06

**Table A6:** Solubility parameter *δ*_2_ of proanthocyanidins at different temperatures ((J/cm^−3^)^0.5^) (obtained by IGC).

Larch Bark Proanthocyanidins	323.15 K	333.15 K	343.15 K	353.15 K	363.15 K	373.15 K	383.15 K	298.15 K
LPPCs	19.16	18.86	18.66	18.17	17.57	16.94	16.52	20.50
LOPCs	20.74	20.58	20.06	19.17	19.02	-	-	22.09

**Table A7:** Solubility parameters of proanthocyanidins simulated by molecular dynamics at 298.15K with different degrees of polymerization.

Proanthocyanidins	$\delta_{elec}/(J\cdot {\mathrm{cm}}^{-3}{)}^{0.5}$	$\delta_{vdw}/(J\cdot {\mathrm{cm}}^{-3}{)}^{0.5}$	$\delta_{total}/(J\cdot {\mathrm{cm}}^{-3}{)}^{0.5}$
trimer	16.24	15.03	22.35
heptamer	14.26	14.51	20.57

**Table A8:** Dispersion component, specific component and total surface energy of LPPCs and LOPCs at different temperatures.

T/K	$\gamma_{s}^{d}$	$\gamma_{s}^{-}$	$\gamma_{s}^{+}$	$\gamma_{s}^{sp}$	$\gamma_{s}$
LPPCs					
343.15	47.15	2.32	10.66	9.95	57.10
353.15	43.26	0.75	8.76	5.14	48.39
363.15	41.47	1.92	5.00	6.19	47.66
373.15	41.12	2.24	5.12	6.77	47.90
383.15	39.26	1.60	7.17	6.77	46.03
LOPCs					
323.15	46.06	2.20	0.04	0.56	46.65
333.15	42.80	1.71	1.47	3.17	45.97
343.15	40.92	1.07	2.88	3.51	44.44
353.15	39.19	0.01	1.02	0.18	39.37
363.15	36.50	0.01	2.01	0.28	36.78

**Table A9:** Surface specific adsorption free energy $∆G^{sp}$ (kJ/mol) of LPPCs and LOPCs at different temperatures.

LPPCs					
Probe solvent	343.15 K	353.15 K	363.15 K	373.15 K	383.15 K
Chloroform	1.88	1.52	0.93	0.56	0.67
THF	6.48	5.42	4.14	3.86	3.88
Ethyl acetate	6.00	5.70	3.10	2.43	2.23
**LOPCs**					
Probe solvent	323.15 K	333.15 K	343.15 K	353.15 K	363.15 K
Chloroform	0.80	0.96	1.11	0.38	0.88
THF	5.43	5.51	5.64	4.62	4.26
Ethyl acetate	3.85	3.86	4.19	3.48	2.94
